# Split or full-body workout routine: which is best to increase muscle strength and hypertrophy?

**DOI:** 10.31744/einstein_journal/2021AO5781

**Published:** 2021-08-18

**Authors:** Alexandre Lopes Evangelista, Tiago Volpi Braz, Cauê Vazquez La Scala Teixeira, Roberta Luksevicius Rica, Angélica Castilho Alonso, Welmo Alcântara Barbosa, Victor Machado Reis, Julien Steven Baker, Brad Jon Schoenfeld, Danilo Sales Bocalini, Julia Maria D’Andréa Greve

**Affiliations:** 1 Universidade Nove de Julho São PauloSP Brazil Universidade Nove de Julho, São Paulo, SP, Brazil.; 2 Universidade Metodista de Piracicaba PiracicabaSP Brazil Universidade Metodista de Piracicaba, Piracicaba, SP, Brazil.; 3 Universidade Federal de São Paulo SantosSP Brazil Universidade Federal de São Paulo, Santos, SP, Brazil.; 4 Universidade São Judas Tadeu São PauloSP Brazil Universidade São Judas Tadeu, São Paulo, SP, Brazil.; 5 Universidade Federal do Espírito Santo VitóriaES Brazil Universidade Federal do Espírito Santo, Vitória, ES, Brazil.; 6 Centro de Investigação em Desporto, Saúde e Desenvolvimento Humano Vila Real Portugal Centro de Investigação em Desporto, Saúde e Desenvolvimento Humano, Vila Real, Portugal.; 7 Hong Kong Baptist University Kowloon Tong China Hong Kong Baptist University, Kowloon Tong, China.; 8 Lehman College BronxNY United States Lehman College, Bronx, NY, United States.; 9 Universidade de São Paulo Faculdade de Medicina Hospital das Clínicas São PauloSP Brazil Instituto de Ortopedia e Traumatologia, Hospital das Clínicas, Faculdade de Medicina, Universidade de São Paulo, São Paulo, SP, Brazil.

**Keywords:** Resistance training, Muscle development/physiology, Muscle, skeletal/growth & development, Muscle strength, Hypertrophy

## Abstract

**Objective:**

To compare the effects of different resistance training programs on measures of muscle strength and hypertrophy.

**Methods:**

Sixty-seven untrained subjects were randomized to one of two groups: Split Workout Routine (n=35), in which muscle groups were trained twice per week in an A/B split consisting of eight sets per session, or Full-Body Workout Routine (n=32), in which muscle groups were trained four times per week with four and eight sets per session. Both groups performed eight to 12 repetition maximum per set, with 60 seconds of rest between sets. Maximal strength and muscle thickness were assessed at baseline and after eight weeks of training.

**Results:**

A significant main effect of time (pre *versus* post) was observed for maximal strength in the bench press and squat exercises and thickness of the elbow extensor, elbow flexor and *quadriceps femoris* muscles. Selected variables did not differ significantly between groups.

**Conclusion:**

Resistance training twice or four times per week has similar effects on neuromuscular adaptation, provided weekly set volume is equal.

## INTRODUCTION

Resistance training (RT) is widely known as the most effective way to increase muscle strength and mass, *i.e.,* muscle hypertrophy in humans.^(^^1^^)^ Maximization of muscle adaptation requires proper manipulation of RT variables. One variable of particular interest is RT frequency. Frequency is sometimes defined as the number of training sessions per week. However, the number of times a given muscle group is trained per week is a more common definition.

In a recent meta-analysis, hypertrophic gains were associated with training muscle groups more than once per week.^(^^2^^)^ However, it is not clear whether higher weekly training frequencies translate into superior gains relative to lower frequencies. The paucity of studies addressing higher frequency of training may stem from the premise that a minimum of 48 hours of recovery between training sessions targeting the same muscle groups is needed, *as per* guidelines published by the American College of Sports Medicine (ACSM).^(^^3^^)^

Dankel et al.,^(^^4^^)^ recently challenged the ideia that muscles require a minimum of 48 hours to recover from a training session. The research hypothesis in that study was that the combination of lower training volume and higher frequency would help to increase the area under the curve of muscle protein synthesis response, leading to greater muscle mass gains over time. Plausibility aside, few studies to date have tested this hypothesis in young sedentary individuals.

## OBJECTIVE

To compare the effects of different resistance training programs on muscle strength and hypertrophy measures.

## METHODS

### Participants

Eighty-six healthy young men volunteered to participate in this study. Participants were assigned to Split Workout Routine and Full-Body Workout Routine Groups using a computer-based random number generator. Randomization occurred within blocks of six subjects. In each block, two subjects were allocated to each group in order to achieve a 1:1 recruitment balance throughout the study. A sample size of 16 subjects per group was determined according to a prior study.^(^^5^^)^

Exclusion criteria were as follows: strength training background for the last 6 months, clinical diagnosis of *diabetes mellitus*, being a smoker, musculoskeletal complications and/or cardiovascular changes confirmed by medical evaluation. Nineteen participants dropped out of the study for personal reasons. The final sample comprised 67 participants, who were randomized to one of two groups: Split Workout Routine (n=35; age: 26.2±4.6 years; height: 1.69±0.07mts; body mass: 69.9±9kg) or Full-Body Workout Routine (n=32; age: 27.5±7.6 years; height: 1.7±0.08mts; body mass: 72.5±13.9kg).

The experimental period consisted of 10 weeks. The first week corresponded to familiarization and pre-intervention period (baseline); during the second to ninth week, we had the training intervention period; and the tenth week was the postintervention period.

Participants in this study read and signed an informed consent form. This study was approved by the Ethics and Research Committee (CAAE: 83122818.0.0000.5511, protocol number 2.549.504/2018) of and conducted at *Universidade Nove de Julho*.

### Maximal strength assessment

Maximal dynamic strength assessment was based on the one repetition maximum (1RM) test using the bench press (1R_MBENCH PRESS_) and the squat (1RM_SQUAT_) exercises. Specifically the 1RM_SQUAT_ was performed with 90° of amplitude using a smith machine equipment. Maximal dynamic strength was measured at baseline and at the end of the study. The test protocol was designed according to recommendations given elsewhere.^(^^6^^)^ Prior to baseline assessment, subjects were instructed to refrain from exercise other than activities of daily living for a minimum of 72 hours. Similar instructions were given prior to the final assessment.

In brief, participants performed a general warm-up consisting of a 5-minute walk on a treadmill (Movement Technology, São Paulo, Brazil) at 60% of maximum heart rate. This was followed by two specific warm-upsets with proper lifting technique. The first set consisted of five repetitions at ~50% of the estimated 1RM weight. A second set of three repetitions with loads corresponding to ~60 to 80% of the estimated 1RM weight was then executed. Subjects were allowed a 3-minute rest period between sets. Finally, subjects had five attempts at 1RM with a 3-minute rest period between trials. 1RM was operationally defined as the maximum weight that could be lifted a single time with proper technique. Verbal encouragement was given throughout the 1RM test session. Sessions were supervised by the research team to ensure safety and validity of attempts.

### Muscle thickness assessment

Ultrasound imaging was used to measure the muscle thickness (MT) at baseline and at the end of the study. A trained technician performed all measurements using B-mode ultrasonography (Mindray; DP10; Shenzhen, China). Following application of generous amounts of water-soluble transmission gel (Mercur S.A., Body Care, Santa Cruz do Sul, RS, Brazil) to the measurement site, a 7.5 to 10MHz linear probe was positioned perpendicular to the target muscle without compressing the skin. Settings were selected according to manufacturer’s specifications for optimal image quality and kept constant throughout. Satisfactory images were saved to a computer hard drive and MT estimated by measuring the distance from the subcutaneous adipose-muscle tissue interface to the muscle-bone interface, as previously described.^(^^7^^)^Measurements were made on the right side of the body at 4 sites, as follows: *biceps brachii* (MT_BB_), t*riceps brachii* (MT_TB_), *vastus lateralis* (MT_VL_) and *rectus femoris* (MT_RF_). Upper arm and lower limb measurements were made with participants in the standing and the supine position respectively. On the anterior and posterior aspects of the upper arm, measurements were made at 60% of the distance between the lateral epicondyle of the humerus and the acromion process of the scapula. Thigh muscle measurements were made at 50% of the distance between the lateral condyle of the femur and the greater trochanter. The limb was secured to minimize unwanted movement during measurements. Anatomical landmarks were marked with henna dye to ensure consistency between pre-and post-intervention measurements. Markings were touched up weekly.

Images were obtained 48 to 72 hours after the final training session to avoid potential interferences of post-workout muscle swelling with results. Research has shown acutely increased MT to return to baseline within 48 hours of RT.^(^^8^^)^ To further ensure the accuracy of measurements, a minimum of three images were obtained per site. Measurements differing by less than 1mm were averaged to a final value. Whenever measurements differed by more than 1mm, a fourth image was obtained and the three closest measurements averaged.

The intraclass correlation coefficient (ICC) for test-retest for MT_BB_, MT_TB_, MT_RF_ and MT_VL_ was 0.998 (95% confidence interval – 95%CI: 0.986-0.999), 0.996 (95%CI: 0.981-0.999), 0.999 (95%CI: 0.972-0.999), and 0.995 (95%CI: 0.980-0.998), respectively. The standard error of the mean (SEM) for these measures was 0.42mm (95%CI: 0.22-0.62mm), 0.29mm (95%CI: 0.12-0.47mm), 0.41mm (95%CI: 0.09-0.73mm) and 0.52mm (95%CI: 0.33-0.71mm), to MT_BB_, MT_TB_, MT_RF_ and MT_VL_ respectively. These values were calculated from the three images captured per site.

### Exercise training design

Sixty-seven untrained subjects were randomized to one of two groups: Split Workout Routine (n=35), in which different muscle groups were trained twice weekly in an A/B split consisting of eight sets per session, or Full-Body Workout Routine (n=32), in which all muscle groups were trained four times weekly with four sets per session. Following technical familiarization, both groups completed four weekly sessions.

The only difference between groups was the frequency with which each muscle group was trained. Split Workout Routine training comprised an ‘A’ (Mondays and Thursdays) and a ‘B’ (Tuesdays and Fridays) session. The A session consisted of bench press, inclined bench press, cable triceps pushdown, triceps kickback, shoulder press and front dumbbell raise. The B session consisted of seated row, lat pulldown, biceps curl, hammer curl, squat and leg curl. In contrast, the Full-Body Workout Routine trained each muscle group four times per week (Mondays, Tuesdays, Thursdays and Fridays) with the following exercises: bench press, cable triceps pushdown, shoulder press, seated row, biceps curl, squat and leg curl. Subjects reported a rating of perception exertion of 9.5 to 10 for all sets and exercises across RT sessions based on the rating of perception exertion.^(^^9^^)^ Specific Split Workout Routine and Full-Body Workout Routine programs are described in table 1.

**Table 1:** Training program design for Split Workout Routine and Full-Body Workout Routine

Workout routine	Monday	Tuesday	Wednesday	Thursday	Friday	Total of sets per week (per muscle group)
Split	A	B		A	B	16
	Bench press 4x8-12 RM	Seated row 4x8-12 RM		Bench press 4x8-12 RM	Seated row 4x8-12 RM	
	Inclined bench press 4x8-12 RM	Lat pulldown 4x8-12 RM		Inclined bench Press 4x8-12 RM	Lat pulldown 4x8-12 RM	
	Cable triceps pushdown 4x8-12 RM	Biceps curl 4x8-12 RM		Cable triceps pushdown 4x8-12 RM	Biceps curl 4x8-12 RM	
	Triceps kickback 4x8-12 RM	Hammer curl 4X8-12 RM		Triceps kickback 4x8-12 RM	Hammer curl 4x8-12 RM	
	Shoulder press 4x8-12 RM	Squat 8x8-12 RM		Shoulder press 4x8-12 RM	Squat 8x8-12 RM	
	Front dumbbell raise 4x8-12 RM	Leg curl 8x8-12 RM		Front dumbbell raise 4x8-12 RM	Leg curl 8x8-12 RM	
Full-body	Bench press 4x8-12 RM	Bench press 4x8-12 RM		Bench press 4x8-12 RM	Bench press 4x8-12 RM	16
	Cable triceps pushdown 4x8-12 RM	Cable triceps pushdown 4x8-12 RM		Cable triceps pushdown 4x8-12 RM	Cable triceps pushdown 4x8-12 RM	
	Shoulder press 4x8-12 RM	Shoulder press 4x8-12 RM		Shoulder press 4x8-12 RM	Shoulder press 4x8-12 RM	
	Seated row 4x8-12 RM	Seated row 4x8-12 RM		Seated row 4x8-12 RM	Seated row 4x8-12 RM	
	Biceps curl 4x8-12 RM	Biceps curl 4x8-12 RM		Biceps curl 4x8-12 RM	Biceps curl 4x8-12 RM	
	Squat 4x8-12 RM	Squat 4x8-12 RM		Squat 4x8-12 RM	Squat 4x8-12 RM	
	Leg curl 4x8-12 RM	Leg curl 4x8-12 RM		Leg curl 4x8-12 RM	Leg curl 4x8-12 RM	

RM: repetition max.

All sessions were preceded by a specific warm-up consisting of one set of ten repetitions with 50% of the load used in the first set of each exercise. Subjects in both groups then executed eight to 12 RM per set. Subjects allocated to Split Workout Routine or Full-Body Workout Routine executed eight and four sets per muscle group respectively. Exercises were performed at no specific tempo, with a 60-second rest period between sets. Researchers supervised all training sessions and provided verbal encouragement to ensure the subjects executed the required number of sets and repetitions. The weekly- sets volume was equated between groups, attempts were made to progressively increase or decrease of the external loads lifted weekly to maintaining the target repetition range.

### Statistical analysis

Normality and homogeneity of variances were tested using the Shapiro-Wilk test and the Levene test, respectively. Prior to analysis, data were log-transformed to reduce skewness resulting from non-uniformity of error (heteroscedasticity). Means±standard deviations (SD) and 95%CI were calculated following confirmation of data normality. Repeated measures analysis of variance (Anova) was used to compare 1RM_BENCH PRESS_, 1RM_SQUAT_, MT_BB_, MT_TB_, MT_RF_, MT_VL_ and the effects of time (pre- *versus* post-intervention) between groups (Split Workout Routine *versus* Full-Body Workout Routine). Bonferroni post-hoc tests were then used to determine significant differences. Effect size (ES) was also estimated using partial eta squared (η^2^_p_); <0.06, 0.06 to 0.14 and >0.14 indicated small, medium and large effect respectively. Effect size was expressed as the absolute difference (pre *versus* post) in the raw value of variables using the difference between two means in standardized units (Cohen’s d value). Cohen’s d values were qualitatively interpreted according to the following thresholds: <0.2, trivial; 0.2 to 0.6, small; 0.6 to 1.2, moderate; 1.2 to 2.0, large; 2.0 to 4.0, very large and >4.0, nearly perfect. Whenever 90% confidence intervals overlapped, small positive and negative values attributed to magnitude were deemed unclear. Otherwise, the magnitude was deemed to be the observed magnitude. Statistical analyses were conducted using (SPSS), version 22.0 software (IBM Corp., Armonk, NY, USA). The level of significance was set at p≤0.05.

## RESULTS

**Figure 1:**
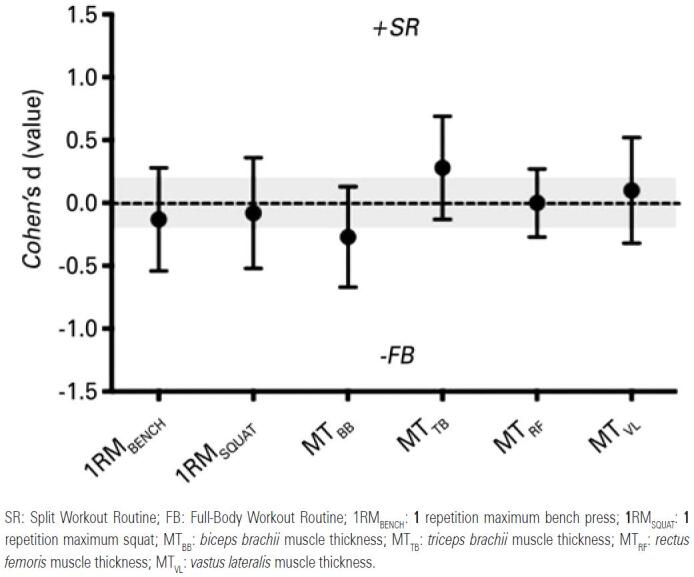
Effect size differences (Cohen’s d) in maximal strength in bench press, squat and thickness of the *biceps Brachii, triceps brachii*, *rectus femoris* and *vastus lateralis* muscles between groups

SR: Split Workout Routine; FB: Full-Body Workout Routine; 1RM_BENCH_: **1** repetition maximum bench press; **1**RM_SQUAT_: **1** repetition maximum squat; MT_BB_: *biceps brachii* muscle thickness; MT_TB_: *triceps brachii* muscle thickness; MT_RF_: *rectus femoris* muscle thickness; MT_VL_: *vastus lateralis* muscle thickness.

Baseline measurements did not differ significantly (p>0.05) between groups. Cohen’s d values reported for all outcomes are shown in figure 1 are expressed as effect size ± 90% confidence interval for comparison of absolute differences between the raw value of variables. The grey area corresponds to trivial differences.

### Maximal strength

There was a significant main effect of time (F_1,31_=280.841; p<0.001; η^2^_p_=0.901) but no group-by-time interaction (F_1,31_=0.012; p=0.914; η^2^_p_=0.001) for 1RM_BENCH PRESS_. A significant increase in 1RM_BENCH PRESS_ from baseline to post-intervention was detected in both the Split Workout Routine (10.5kg; 18.1%; p=0.001; ES=0.47) and the Full-Body Workout Routine (11.2kg; 17.5%; p=0.001; ES=0.38) group (Table 2).

**Table 2:** Muscle strength pre and post after Split Workout Routine or Full-Body Workout Routine

Parameters		Pre	Post	Anova					
				Time effect			Time x group effect		
				F	p	η^2^_p_	F	p	η^2^_p_
1RM_BENCH PRESS_, kg									
	SR	56.4±20.9	67.4±21.8*	280.841	0.001	0.901	0.012	0.914	0.001
	FB	63.8±28.9	74.9±29.9*						
1RM_SQUAT_, kg									
	SR	85.5±26.6	109.5±26.7*	159.770	0.001	0.838	0.412	0.914	0.013
	FB	89.8±30.2	115.4±32.5*						

Values expressed as means±standard deviations for Split Workout Routine and Full-Body Workout Routine Groups.

* Significantly greater than the corresponding pre-intervention value (p<0.05).

Anova: analysis of variance; RM_BENCH_: 1 repetition maximum bench press; SR: Split Workout Routine; FB: Full-Body Workout Routine; 1RM_SQUAT_: 1 repetition maximum squat.

There was a significant main effect of time (F_1,31_=159.770; p<0.001; η^2^_p_=0.838) but no group-by-time interaction (F_1,31_=0.412; p=0.914; η^2^_p_=0.013) for 1RM_SQUAT_. A significant increase in 1RM_SQUAT_ from baseline to post-intervention was detected in both the Split Workout Routine (24.5kg; 28.2%; p=0.001; ES=0.92) and the Full-Body Workout Routine (25.7kg; 28.6%; p=0.001; ES=0.82) group (Table 2).

### Muscle thickness

There was a significant main effect of time (F_1,31_=92.444; p<0.001; η^2^_p_=0.841) but no group-by-time interaction (F_1,31_=0.049; p=0.804; η^2^_p_=0.017) for MT_BB_. *Biceps brachii* MT increased significantly from baseline to post-intervention in both the Split Workout Routine (2.9mm; 9.1%; p=0.001; ES=0.48) and the Full-Body Workout Routine (3.5mm; 11.1%; p=0.001; ES=0.50) group (Table 3).

**Table 3:** Muscle thickness prior to and after Split Workout Routine and Full-Body Workout Routine

Parameters		Pre	Post	Anova					
				Time effect			Time x group effect		
				F	p	η^2^_p_	F	p	η^2^_p_
MT_BB_, mm									
	SR	31±5.8	33.9±5.8*	92.444	0.001	0.841	0.049	0.804	0.017
	FB	31.6±7	35.1±6.9*						
MT_TB_, mm									
	SR	22.1±6.5	26.3±7.2*	156.506	0.001	0.835	1.349	0.254	0.042
	FB	23.9±8.3	27.4±8.3*						
MT_RF_, mm									
	SR	17.7±4.1	19.8±4.1*	86.335	0.001	0.736	0.046	0.832	0.001
	FB	17.9±3.7	20.1±3.7*						
MT_VL_, mm									
	SR	19.1±4.4	21.5±4.6*	97.823	0.001	0.882	0.127	0.813	0.024
	FB	20.4±3.6	22.5±4*						

Values expressed as means±standard deviations for Split Workout Routine and Full-Body Workout Routine Groups.

* Significantly greater than the corresponding pre-intervention value (p<0.05).

Anova: analysis of variance; MT_BB_: *biceps brachii* muscle thickness; SR: Split Workout Routine; FB: Full-Body Workout Routine; MT_TB_: *triceps brachii* muscle thickness; MT_RF_: anterior quadriceps muscle thickness; MT_VL_: *vastus lateralis* muscle thickness.

There was a significant main effect of time (F_1,31_=156.506, p<0.001, η^2^_p_=0.835) but not group-by-time interaction (F_1,31_=1.349; p=0.254; η^2^_p_=0.042) for MT_TB_. *Triceps brachii* MT increased significantly from baseline to post-intervention in both the Split Workout Routine (4.2mm; 18.7%; p=0.001; ES=0.62) and the Full-Body Workout Routine (3.5mm; 14.4%; p=0.001; ES=0.41) group.

There was a significant main effect of time (F_1,31_=86.335; p<0.001; η^2^_p_=0.736) but no group-by-time interaction (F_1,31_=0.046; p=0.832; η^2^_p_=0.001) for MT_RF_. *Rectus femoris* MT increased significantly from baseline to post-intervention in both the Split Workout Routine (2.2mm; 12.3%; p=0.001; ES=0.54) and the Full-Body Workout Routine (2.2mm; 12.1%; p=0.001; ES=0.58) group.

There was a significant main effect of time (F_1,31_=97.823; p<0.001; η^2^_p_=0.882) but no group-by- time interaction (F_1,31_=0.127; p=0.813; η^2^_p_=0.024) MT_VL_. *Vastus lateralis* MT increased significantly from baseline to post-intervention in both the Split Workout Routine (2.3mm; 12.1%; p=0.001; ES=0.51) and the Full-Body Workout Routine (2.1mm; 10.5%; p=0.001; ES=0.56) group.

## DISCUSSION

Individuals in the Split Workout Routine and the Full-Body Workout Routine Groups experienced similar maximal strength gains from baseline to post-intervention. Changes in 1RM_BENCH PRESS_ (18.1% and 17.5% for Split Workout Routine and Full-Body Workout Routine Group, respectively) and 1RM_SQUAT_ (28.2% and 28.6% for Split Workout Routine and Full-Body Workout Routine Group, respectively) were almost identical. Effect sizes for 1RM_BENCH PRESS_ (0.47 and 0.38 for Split Workout Routine and Full-Body Workout Routine Group, respectively) and 1RM_SQUAT_ (0.92 and 0.82 for Split Workout Routine and Full-Body Workout Routine Group, respectively) were also very similar between groups. These findings are in keeping with the current body of literature. Research suggests strength gains derived from frequency manipulation are driven by the increase in training volume. When training volume is held constant, increased frequency does not seem to provide additional benefits.^(^^10^^)^ However, most studies to date employed RT frequencies of 3 or fewer days per muscle group per week. Findings of this study add to the existing literature in that they show RT 4 days per week provides no additional strength gains relative to RT twice per week.

Split Workout Routines are thought to enhance the ability to train at maximal effort level for a given intensity, generating higher muscle strain in a specific training session.^(^^11^^)^ Such workout routines arguably facilitate recovery, since alternating between muscle groups allows more time for a given muscle to recover between training sessions. However, results of this study suggest Split Workout Routine training does not enhance muscle strength adaptations in untrained males compared to Full-Body Workout Routine training, provided volume and intensity are equal. Similar findings have been reported in prior studies with resistance-trained males.^(^^12^^,^^13^^)^

With regard to hypertrophic adaptations, Split Workout Routine and Full-Body Workout Routine led to similar increases in upper and lower limb muscle mass. Volume is thought to be a major factor in hypertrophic adaptation.^(^^14^^,^^15^^)^ Hence, these findings may have reflected equal total training volume in both workout routines. Results of this study are consistent with other studies comparing volume-equated muscle training with frequencies of least 2 days per week.^(^^16^^,^^17^^)^ In the study conducted by Brigatto et al.,^(^^18^^)^ ultrasound measurements revealed no superior hypertrophic gains following training twice *versus* 4 times per week. Brigatto et al.,^(^^18^^)^ employed Split Workout Routines whereas our study compared a Split Workout Routine and Full-Body Workout Routine.

Zaroni et al.,^(^^19^^)^ also compared the effects Split Workout Routine and Full-Body Workout Routine on neuromuscular adaptation in well-trained men over the course of eight weeks of training. In that study, muscle groups were trained once (Split) or five times (Total) per week and subjects executed the same exercises with similar repetition ranges each training week throughout the experimental period. Different from this study, higher weekly training frequency was associated with potentially higher hypertrophic effects in subjects submitted to a Split Workout Routine inwhich muscles were trained only once per week (in spite of equalized volume). However, the sample in that study comprised well-trained individuals (RT experience=2.4 to 6.4 years), which may in part explain outcome differences.

Further studies comparing different RT frequencies are obviously needed for deeper understanding of the impact of training frequency on long-term muscle growth.

Some limitations of this study must be pointed. The experimental period was limited to eight weeks without energy intake control. The gain in muscle strength in the present study was probably due to neural adaptations, mainly because to the characteristics of the subjects in the sample, who were untrained. In spite of significant muscle strength and hypertrophy gains in both groups, it is not clear whether prolonged training would have yielded different results. The MT measurements were made at the middle portion of the muscles. This region is often used as a proxy for overall growth of a given muscle. However, research suggests hypertrophy is a region-specific phenomenon and greater gains are sometimes detected at the proximal and distal aspects.^(^^20^^,^^21^^)^ Finally, findings are specific to healthy male subjects and therefore cannot be generalized to other populations, including adolescents, women and elderly and trained individuals. Higher RT frequencies may not be as well tolerated by these individuals and may accelerate the onset of overtraining if combined with high training intensities. Future research is warranted to determine frequency-related responses to RT with energy intake monitoring as well as controled repetitions in this populations. Additionally, in our study the volume control was done by equalizing the number of sets in concordance with recommended in previously studies,^(^^22^^)^ however, is possible consider that equalizing the total load lifted (number of series x number of repetitions x lifted load) could be promoted different outcomes, thus more studies should be conducted for further clarification.

Findings of this study have important implications, as they may assist coaches and trainers in designing individualized training programs, which may enhance exercise adherence. Indeed, results presented suggest strength and conditioning professionals can choose between a wide variety of training frequencies in order to optimize muscle strength and hypertrophy for a given weekly volume.

This study was designed to isolate the effects of frequency on muscular adaptation, while remaining workout variables were kept constant. Given the dose-response relationship between training volume and muscular adaptation,^(^^15^^,^^23^^)^ higher training frequency may allow practitioners to handle greater volumes of training, which in turn may result in larger muscle strength and hypertrophy gains. Whether additional training volume resultant from higher training frequency would be ideal or excessive would be unique to each individual.

### Practical applications

Split Workout Routine and Full-Body Workout Routine promoted similar gains in muscle strength and thickness in upper and lower limbs in untrained individuals. Both training strategies are equally effective in untrained individuals during the early phase of training (eight weeks). Findings of this study may enhance exercise adherence by tailoring training programs to individual needs. Strength and conditioning professionals can use a wide range of training frequencies to optimize muscle strength and hypertrophy for a given weekly training volume.

## CONCLUSION

Split Workout Routine and Full-Body Workout Routine promoted similar gains in muscle strength and thickness in upper and lower limbs. These findings suggest both training strategies are equally effective in enhancing muscular adaptation in untrained individuals during the early phase of resistance training (eight weeks).
