# Supplementary material for: Split or full-body workout routine: which is best to increase muscle strength and hypertrophy?
Source: Einstein (Sao Paulo). 2021 Aug 18;19:eAO5781. doi: 10.31744/einstein_journal/2021AO5781 (PMC8372753; doi:10.31744/einstein_journal/2021AO5781)
Supplement: Translation

# Translation: Rotina de treinamento distribuída ou de corpo inteiro: qual a melhor estratégia para aumentar força e hipertrofia muscular?

**Authors:** Alexandre Lopes Evangelista, Tiago Volpi Braz, Cauê Vazquez La Scala Teixeira, Roberta Luksevicius Rica, Angélica Castilho Alonso, Welmo Alcântara Barbosa, Victor Machado Reis, Julien Steven Baker, Brad Jon Schoenfeld, Danilo Sales Bocalini, Julia Maria D’Andréa Greve

**Affiliations:** 1 São PauloSP Brasil Universidade Nove de Julho, São Paulo, SP, Brasil.; 2 PiracicabaSP Brasil Universidade Metodista de Piracicaba, Piracicaba, SP, Brasil.; 3 SantosSP Brasil Universidade Federal de São Paulo, Santos, SP, Brasil.; 4 São PauloSP Brasil Universidade São Judas Tadeu, São Paulo, SP, Brasil.; 5 VitóriaES Brasil Universidade Federal do Espírito Santo, Vitória, ES, Brasil.; 6 Vila Real Portugal Centro de Investigação em Desporto, Saúde e Desenvolvimento Humano, Vila Real, Portugal.; 7 Kowloon Tong China Hong Kong Baptist University, Kowloon Tong, China.; 8 BronxNY United States Lehman College, Bronx, NY, United States.; 9 São PauloSP Brasil Instituto de Ortopedia e Traumatologia, Hospital das Clínicas, Faculdade de Medicina, Universidade de São Paulo, São Paulo, SP, Brasil.

**Keywords:** Treinamento de resistência, Desenvolvimento muscular/fisiologia, Músculo esquelético/crescimento & desenvolvimento, Força muscular, Hipertrofia

## Abstract

**Objetivo:**

Comparar os efeitos de diferentes rotinas de treinamento em medidas de força e hipertrofia muscular.

**Métodos:**

Foram distribuídos em dois grupos de treinamento 67 indivíduos não treinados: Rotina Distribuída (n=35), com os grupos musculares treinados duas vezes por semana em uma divisão A/B com oito séries por sessão, ou Rotina de Corpo Inteiro (n=32), com os grupos musculares treinados quatro vezes por semana com quatro e oito séries por sessão de treinamento. Ambos os grupos realizaram de oito a 12 repetições máximas por série, com intervalo de 60 segundos entre as séries. A força e a espessura muscular máximas foram avaliadas no início e após oito semanas de treinamento.

**Resultados:**

Observaram-se diferenças significativas considerando o tempo (pré *versus* pós) para força máxima no supino, agachamento e espessura muscular de extensores do cotovelo, flexores do cotovelo e músculos do quadríceps femoral. Não foram observadas diferenças significativas entre os grupos para nenhum dos parâmetros avaliados.

**Conclusão:**

O treinamento de força realizado duas ou quatro vezes por semana com volume de séries equalizado promove adaptações neuromusculares similares.

## INTRODUÇÃO

O treinamento de força (TF) é amplamente conhecido como o meio mais eficaz de aumentar a força e a massa muscular, ou seja, promover a hipertrofia muscular em seres humanos.^(^^1^^)^ A maximização da adaptação muscular requer a manipulação adequada das variáveis do TF, sendo a frequência uma variável de particular interesse. Frequência pode ser definida como o número de sessões semanais de treinamento, embora o número de vezes que um determinado músculo é treinado por semana seja uma definição mais comum.

Em metanálise recente, ganhos hipertróficos foram associados ao treinamento de grupos musculares mais de uma vez por semana.^(^^2^^)^ Entretanto, não se sabe ao certo se treinamentos semanais mais frequentes proporcionam melhores resultados do que treinamentos menos frequentes. A escassez de estudos que abordam frequências mais altas de treinamento pode refletir a premissa de que um período de recuperação mínimo de 48 horas entre sessões que trabalham um mesmo músculo se faz necessário, conforme publicado nas diretrizes do *American College of Sports Medicine* (ACSM).^(^^3^^)^

Recentemente, Dankel et al.,^(^^4^^)^ desafiaram o conceito de que um músculo necessita de um mínimo de 48 horas para se recuperar de uma sessão de treinamento. O estudo em questão levantou a hipótese de que a combinação de menor volume e maior frequência de treinamento ajudaria a aumentar a área sob a curva no que se refere à resposta de síntese de proteína muscular, proporcionando maior ganho de massa muscular ao longo do tempo. Embora plausível, poucos estudos até hoje testaram essa hipótese em jovens sedentários.

## OBJETIVO

Comparar os efeitos de diferentes programas de treinamento de força sobre parâmetros de força e hipertrofia muscular.

## MÉTODOS

### Participantes

Voluntariaram-se para participar deste estudo 86 homens jovens e saudáveis. Os participantes foram designados para os Grupos Rotina Distribuída ou Rotina de Corpo Inteiro, por meio de um gerador computadorizado de números aleatórios. A randomização foi realizada em blocos de seis participantes. Em cada bloco, dois participantes foram alocados para cada grupo, a fim de proporcionar um recrutamento equilibrado de 1:1 ao longo do estudo. Uma amostra de 16 indivíduos por grupo foi considerada necessária, com base em estudo prévio.^(^^5^^)^

Foram adotados os seguintes critérios de exclusão: experiência em TF nos últimos 6 meses, diagnóstico clínico de *diabetes mellitus*, tabagismo, complicações musculoesqueléticas e/ou alterações cardiovasculares confirmadas por exame médico. Dezenove participantes se desligaram do estudo por motivos pessoais. A amostra final incluiu 67 participantes, que foram divididos aleatoriamente em dois grupos: Rotina Distribuída (n=35; idade: 26,2±4,6 anos; altura: 1,69±0,07mts; massa corporal: 69,9±9kg) ou Rotina de Corpo Inteiro (n=32; idade: 27,5±7,6 anos; altura: 1,7±0,08mts; massa corporal: 72,5±13,9kg).

O período experimental foi de 10 semanas. A primeira semana correspondeu a um período de familiarização e pré-intervenção (linha de referência); da segunda à nona semana, houve um período de intervenção/treinamento; a décima semana foi o período pós-intervenção.

Todos os participantes leram e assinaram um Termo de Consentimento informado. Este estudo foi aprovado pelo Comitê de Ética e Pesquisa (CAAE: 83122818.0.0000.5511, parecer 2.549.504/2018) e conduzido na Universidade Nove de Julho.

### Avaliação de força máxima

A avaliação da força máxima dinâmica foi baseada no teste de uma repetição máxima (1RM) nos exercícios supino (1RM_SUPINO_) e agachamento (1RM_AGACHAMENTO_). O teste de 1RM_AGACHAMENTO_ foi realizado com 90 graus de amplitude usando o *smith machine*. A força máxima dinâmica foi mensurada no início e final do estudo. O protocolo de teste foi criado com bases em recomendações disponibilizadas na literatura.^(^^6^^)^ Antes do teste inicial, os participantes foram instruídos a não realizar exercícios além das atividades cotidianas por um mínimo de 72 horas. As mesmas instruções foram repetidas antes do teste final.

Resumidamente, os participantes realizaram um aquecimento geral correspondente a uma caminhada de 5 minutos em esteira (Movement Technology, São Paulo, Brasil) a 60% da frequência cardíaca máxima, seguido de duas séries específicas de aquecimento executada com técnica adequada. A primeira série foi executada com aproximadamente 50% do peso estimado de 1RM. Seguiu-se uma segunda série de três repetições com cargas correspondentes a cerca de 60% a 80% do peso estimado de 1RM. O período de descanso entre as séries foi de 3 minutos. Finalmente, os participantes tiveram direito a cinco tentativas de 1RM com 3 minutos de descanso entre os ensaios. A definição operacional de 1RM foi o peso máximo levantado uma única vez com a técnica correta. Os participantes receberam estímulo verbal ao longo de toda a sessão de teste de 1RM. As sessões foram supervisionadas pelos pesquisadores, para garantir a segurança e a validade das tentativas.

### Avaliação da espessura muscular

A espessura do músculo (EM) foi mensurada por ultrassonografia no início e no final do estudo. Um técnico treinado realizou todas as mensurações empregando ultrassonografia modo B (Mindray; DP10; Shenzhen, China). Após a aplicação de quantidade generosa de gel de transmissão (Mercur S.A., Body Care, Santa Cruz do Sul, RS, Brasil) sobre o local de medição, um transdutor linear de 7.5 a 10MHz foi posicionado perpendicularmente sobre o músculo, sem comprimir a pele. Os ajustes foram selecionados de acordo com as recomendações do fabricante para qualidade ideal de imagem e mantidos inalterados. As imagens consideradas satisfatórias foram salvas em disco rígido, e a EM foi determinada por meio da mensuração da distância entre a interface tecido adiposo-tecido muscular e a interface tecido muscular-osso, conforme descrição prévia.^(^^7^^)^ As seguintes mensurações foram realizadas do lado direito do corpo: bíceps braquial (EM_BB_), tríceps braquial (EM_TB_), vasto laterial (EM_VL_) e reto femoral (EM_RF_). As mensurações do membro superior e inferior foram realizadas com os participantes em pé e na posição supinada, respectivamente. As mensurações nas faces anterior e posterior do braço foram realizadas a 60% da distância entre o epicôndilo lateral do úmero e o acrômio. As mensurações dos músculos da coxa foram realizadas a 50% da distância entre o côndilo lateral do fêmur e o trocânter maior. O membro foi contido para minimizar a movimentação indesejável durante as mensurações. Os pontos de medição foram marcados com henna para garantir a repetibilidade antes e após a intervenção. As marcações foram retocadas semanalmente.

As imagens foram adquiridas entre 48 e 72 horas após a última sessão de treinamento, a fim de evitar possíveis interferências do aumento de volume muscular pós-treino nos resultados. Foi demonstrado que o aumento agudo da EM se dissipa em 48 horas após o TF.^(^^8^^)^ Para maior acurácia das mensurações, foram obtidas pelo menos três imagens de cada local. A média das mensurações que diferiram em menos de 1mm foi calculada para gerar um valor final. No caso de mensurações que diferiram em mais de 1mm, uma quarta imagem foi obtida, e as três mensurações mais próximas foram empregadas para cálculo da média.

O coeficiente de correlação intraclasse (CCI) teste-reteste para EM_BB_, EM_TB_, EM_RF_ e EM_VL_ foi de 0,998(intervalo de confiança de 95% – IC95%: 0,986-0,999), 0,996 (IC95%: 0,981-0,999), 0,999 (IC95%: 0,972-0,999) e 0,995 (IC95%: 0,980-0,998), respectivamente. O erro padrão da media (EPM) dessas medidas foi de 0,42mm (IC95%: 0,22-0,62mm), 0,29mm (IC95%: 0,12-0,47mm), 0,41mm (IC95%: 0,09-0,73mm) e 0,52mm (IC95%: 0,33-0,71mm), para EM_BB_, EM_TB_, EM_RF_ e EM_VL_, respectivamente. Esses valores foram calculados a partir das três imagens capturadas por local.

### Programa de treinamento

Sessenta e sete indivíduos destreinados foram aleatoriamente alocados para o Grupo Rotina Distribuída (n=35), no qual diferentes grupos musculares foram treinados duas vezes por semana em um esquema A/B com oito séries por sessão, ou Grupo Rotina de Corpo Inteiro (n=32), no qual todos os grupos musculares foram treinados quatro vezes por semana empregando-se quatro séries por sessão. Após a familiarização técnica, ambos os grupos completaram quatro sessões semanais.

A única diferença entre os grupos foi a frequência com que cada grupo muscular foi treinado. A Rotina Distribuída consistiu de uma sessão ‘A’ (segundas e quintas-feiras) e uma sessão ‘B’ (terças e sextas-feiras). A sessão A incluiu os seguintes exercícios: supino reto, supino inclinado, tríceps cabo, tríceps coice, desenvolvimento e elevação frontal com halteres. A sessão B incluiu os exercícios remada sentada, puxada pela frente (*lat pulldown*), rosca bíceps, rosca martelo, agachamento e flexão de joelhos. Em contrapartida, o Grupo Rotina de Corpo Inteiro treinou cada grupo muscular quatro vezes por semana (segundas, terças, quintas e sextas-feiras) com os seguintes exercícios: supino reto, tríceps cabo, desenvolvimento, remada sentada, rosca bíceps, agachamento e flexão de joelhos. Em todas as sessões, os participantes relataram percepção subjetiva de esforço de 9,5 a 10 em todas as séries e exercícios, com base em uma escala de percepção subjetiva de esforço.^(^^9^^)^ Os programas específicos de Rotina Distribuída e Rotina de Corpo Inteiro encontram-se descritos na tabela 1.

**Tabela 1:** Programas de treinamento distribuído e de corpo inteiro

Sessão	Segunda-feira	Terça-feira	Quarta-feira	Quinta-feira	Sexta-feira	Total de séries por semana (por grupo muscular)
Distribuído	A	B		A	B	16
	Supino reto 4x8-12 RM	Remada sentada 4x8-12 RM		Supino reto 4x8-12 RM	Remada sentada 4x8-12 RM	
	Supino inclinado 4x8-12 RM	Puxada pela frente (*lat pulldown*) 4x8-12 RM		Supino inclinado 4x8-12 RM	Puxada pela frente (*lat pulldown*) 4x8-12 RM	
	Tríceps cabo 4x8-12 RM	Rosca bíceps 4x8-12 RM		Tríceps cabo 4x8-12 RM	Rosca bíceps 4x8-12 RM	
	Tríceps coice 4x8-12 RM	Rosca martelo 4x8-12 RM		Tríceps coice 4x8-12 RM	Rosca martelo 4x8-12 RM	
	Desenvolvimento 4x8-12 RM	Agachamento 8x8-12 RM		Desenvolvimento 4x8-12 RM	Agachamento 8x8-12 RM	
	Elevação frontal com halteres 4x8-12 RM	Flexão de Joelhos 8x8-12 RM		Elevação frontal com halteres 4x8-12 RM	Flexão de joelhos 8x8-12 RM	
Corpo inteiro	Supino reto 4x8-12 RM	Supino reto 4x8-12 RM		Supino reto 4x8-12 RM	Supino reto 4x8-12 RM	16
	Tríceps cabo 4x8-12 RM	Tríceps cabo 4x8-12 RM		Tríceps cabo 4x8-12 RM	Tríceps cabo 4x8-12 RM	
	Desenvolvimento 4x8-12 RM	Desenvolvimento 4x8-12 RM		Desenvolvimento 4x8-12 RM	Desenvolvimento 4x8-12 RM	
	Remada sentada 4x8-12 RM	Remada sentada 4x8-12 RM		Remada sentada 4x8-12 RM	Remada sentada 4x8-12 RM	
	Rosca bíceps 4x8-12 RM	Rosca bíceps 4x8-12 RM		Rosca bíceps 4x8-12 RM	Rosca bíceps 4x8-12 RM	
	Agachamento 4x8-12 RM	Agachamento 4x8-12 RM		Agachamento 4x8-12 RM	Agachamento 4x8-12 RM	
	Flexão de joelhos 4x8-12 RM	Flexão de joelhos 4x8-12 RM		Flexão de joelhos 4x8-12 RM	Flexão de joelhos 4x8-12 RM	

RM: repetição máxima.

Todas as sessões foram precedidas por um aquecimento específico correspondente a uma série de dez repetições com 50% da carga empregada na primeira série de cada exercício. Em seguida, os participantes de ambos os grupos executaram de oito a 12 repetições por série. Os participantes alocados para regime Rotina Distribuída ou Rotina de Corpo Inteiro executaram oito e quatro séries por grupo muscular, respectivamente. Os exercícios foram executados com cadência livre e com 60 segundos de descanso entre as séries. Os pesquisadores supervisionaram todas as sessões de treinamento e utilizaram estímulos verbais para garantir a execução do número necessário de séries e repetições por parte dos participantes.

O volume das séries semanais foi equacionado entre os grupos, foram feitas tentativas de aumentar ou diminuir progressivamente as cargas externas levantadas semanalmente para manter a faixa de repetição alvo.

### Análise estatística

A normalidade e a homogeneidade das variâncias foram testadas empregando-se o teste de Shapiro-Wilk e o teste de Levene, respectivamente. Antes da análise, os dados foram log-transformados, para diminuir a distorção resultante da não uniformidade dos erros (heterocedasticidade). Médias±desvios-padrão (DP) e IC95% foram empregados após a confirmação da normalidade dos dados. A análise de variância (Anova) para medidas repetidas foi empregada para comparar 1RM_SUPINO_, 1RM_AGACHAMENTO_, EM_BB_, EM_TB_, EM_RF_, EM_VL_ e o efeito do tempo (pré e pós-intervenção) entre os Grupos Rotina de Corpo Inteiro e Rotina Distribuída. Em seguida, os testes *post-hoc* de Bonferroni foram empregados para determinação das diferenças significantes. O tamanho do efeito (TE) foi estimado empregando-se o eta-quadrado parcial (η^2^_p_), com <0,06, 0,06 a 0,14 e >0,14 indicando efeito pequeno, médio e grande, respectivamente. O TE foi expresso em diferenças absolutas (pré e pós) entre os valores brutos das variáveis, empregando-se a diferença entre duas médias baseada em unidades padronizada (valor d de Cohen). Os valores d de Cohen foram interpretados qualitativamente segundo os seguintes limiares: <0,2, trivial; 0,2 a 0,6, pequeno; 0,6 a 1,2, moderado; 1,2 a 2,0, grande; 2,0 a 4,0, muito grande e >4,0, quase perfeito. Na presença de sobreposição de intervalos de confiança de 90%, valores positivos e negativos pequenos atribuídos à magnitude foram considerados incertos. Caso contrário, a magnitude observada foi a magnitude considerada. As análises estatísticas foram realizadas por meio do software (SPSS), versão 22.0 (IBM Corp., Armonk, NY, EUA). O nível de significância estabelecido foi de p≤0,05.

## RESULTADOS

As mensurações iniciais não diferiram (p>0,05) entre os grupos. Os valores d de Cohen reportados para todos os desfechos podem ser visualizados na figura 1, sendo expressos na forma de tamanho do efeito ± intervalo de confiança de 90% para comparação das diferenças absolutas entre os valores brutos das variáveis. A área em cinza corresponde a diferenças triviais.

**Figura 1:** Diferenças de tamanho do efeito (d de Cohen) sobre a força máxima no supino, agachamento e espessura dos grupos musculares bíceps braquial, tríceps braquial, reto femoral e vasto lateral

TD: Treinamento Rotina Distribuída; FB: Treinamento Rotina de Corpo Inteiro; 1RM_SUPINO_: 1 repetição máxima no supino; 1RM_AGACHAMENTO_: 1 repetição máxima no agachamento; EM_BB_: espessura do músculo bíceps braquial; EM_TB_: espessura do músculo tríceps braquial; EM_RF_: espessura do músculo reto femoral; EM_VL_: espessura do músculo vasto lateral.

### Força máxima

O tempo teve efeito significante (F_1,31_=280,841; p<0,001; η^2^_p_=0,901) sobre 1RM_SUPINO_, porém não houve interações significantes entre grupo e tempo (F_1,31_=0,012; p=0,914; η^2^_p_=0,001). A carga registrada como 1RM_SUPINO_ aumentou entre a linha de referência e o período pós-intervenção tanto no Grupo Rotina Distribuída (10,5kg; 18,1%; p=0,001; TE=0,47) quanto no Grupo Rotina Corpo Inteiro (11,2kg; 17,5%; p=0,001; TE=0,38) (Tabela 2).

**Tabela 2:** Força muscular antes e depois do Rotina Distribuída ou Rotina de Corpo Inteiro

Parâmetros		Pré	Pós	Anova					
				Efeito do tempo			Efeito do tempo x grupo		
				F	p	η^2^_p_	F	p	η^2^_p_
1RM_SUPINO_,kg									
	TD	56,4±20,9	67,4±21,8*	280,841	0,001	0,901	0,012	0,914	0,001
	FB	63,8±28,9	74,9±29,9*						
1RM_AGACHAMENTO_,kg									
	TD	85,5±26,6	109,5±26,7*	159,770	0,001	0,838	0,412	0,914	0,013
	FB	89,8±30,2	115,4±32,5*						

Valores expressos em médias ± desvio-padrão para os Grupos Rotina Distribuída e Rotina de Corpo Inteiro.

* Significativamente maior do que o valor pré-intervenção correspondente (p<0,05).

Anova: análise de variância; 1RM_SUPINO_: 1 repetição máxima no supino; TD: Treinamento Rotina Distribuída; FB: Treinamento Rotina de Corpo Inteiro; 1RM_AGACHAMENTO_: 1 repetição máxima no agachamento.

O tempo teve efeito significante (F_1,31_=159,770, p<0,001, η^2^_p_=0,838) sobre 1RM_AGACHAMENTO_, porém não houve interações significantes entre grupo e tempo (F_1,31_=0,412; p=0,914; η^2^_p_=0,013). A carga registrada como 1RM_AGACHAMENTO_ aumentou entre a linha de referência e o período pós-intervenção tanto no Grupo Rotina Distribuída (24,5kg; 28,2%; p=0,001; TE=0,92) quanto no Grupo Rotina Corpo Inteiro (25,7kg; 28,6%; p=0,001; TE=0,82) (Tabela 2).

### Espessura muscular

O tempo teve efeito significante (F_1,31_=92,444; p<0,001; η^2^_p_=0,841) sobre EM_BB_, porém não houve interações significantes entre grupo e tempo (F_1,31_=0,049; p=0,804; η^2^_p_=0,017). A EM_BB_ aumentou entre a linha de referência e o período pós-intervenção tanto no Grupo Rotina Distribuída (2,9mm; 9,1%; p=0,001; TE=0,48) quanto no Grupo Rotina de Corpo Inteiro (3,5mm; 11,1%; p=0,001; TE=0,50) (Tabela 3).

**Tabela 3:** Espessura muscular antes e depois do treinamento muscular distribuído e de corpo inteiro

Parâmetros		Pré	Pós	Anova					
				Efeito do tempo			Efeito do tempo x grupo		
				F	p	η^2^_p_	F	p	η^2^_p_
EM_BB_, mm									
	TD	31±5,8	33,9±5,8*	92,444	0,001	0,841	0,049	0,804	0,017
	FB	31,6±7	35,1±6,9*						
EM_TB_, mm									
	TD	22,1±6,5	26,3±7,2*	156,506	0,001	0,835	1,349	0,254	0,042
	FB	23,9±8,3	27,4±8,3*						
EM_RF_, mm									
	TD	17,7±4,1	19,8±4,1*	86,335	0,001	0,736	0,046	0,832	0,001
	FB	17,9±3,7	20,1±3,7*						
EM_VL_, mm									
	TD	19,1±4,4	21,5±4,6*	97,823	0,001	0,882	0,127	0,813	0,024
	FB	20,4±3,6	22,5±4*						

Valores expressos em médias ± desvio-padrão para os Grupos Rotina Distribuída e Rotina de Corpo Inteiro.

* Significativamente maior do que o valor pré-intervenção correspondente (p<0,05).

Anova: análise de variância; EM_BB_: espessura do músculo bíceps braquial; TD: Treinamento Rotina Distribuída; FB: Treinamento Rotina de Corpo Inteiro; EM_TB_: espessura do músculo tríceps braquial; EM_RF_: espessura do músculo reto femoral; EM_VL_: espessura do músculo vasto lateral.

O tempo teve efeito significante (F_1,31_=156,506; p<0,001; η^2^_p_=0,835) sobre EM_TB_, porém não houve interações significantes entre grupo e tempo (F_1,31_=1,349; p=0,254; η^2^_p_=0,042). A EM_TB_ aumentou entre a linha de referência e o período pós-intervenção tanto no Grupo Rotina Distribuída (4,2mm; 18,7%; p=0,001; TE=0,62) quanto no Rotina de Corpo Inteiro (3,5mm; 14,4%; p=0,001; TE=0,41).

O tempo teve efeito significante (F_1,31_=86,335; p<0,001; η^2^_p_=0,736) sobre EM_RF_, porém não houve interações significantes entre grupo e tempo (F_1,31_=0,046; p=0,832; η^2^_p_=0,001). A EM_RF_ aumentou entre a linha de referência e o período pós-intervenção tanto no Grupo Rotina Distribuída (2,2mm; 12,3%; p=0,001; TE=0,54) quanto no Rotina de Corpo Inteiro (2,2mm; 12,1%; p=0,001; TE=0,58).

O tempo teve efeito significante (F_1,31_=97,823; p<0,001; η^2^_p_=0,882) sobre EM_VL_, porém não houve interações significantes entre grupo e tempo (F_1,31_=0,127; p=0,813; η^2^_p_=0,024). A EM_VL_ aumentou entre a linha de referência e o período pós-intervenção tanto no Grupo Rotina Distribuída (2,3mm; 12,1%; p=0,001; TE=0,51) quanto no Rotina de Corpo Inteiro (2,1mm; 10,5%; p=0,001; TE=0,56).

## DISCUSSÃO

Os indivíduos dos Grupos Rotina de Corpo Inteiro e Rotina Distribuída tiveram ganho semelhante de força máxima entre os período pré e pós-intervenção. As alterações de carga registradas para 1RM_SUPINO_ (18,1% e 17,5% para Grupo Rotina Distribuída e Grupo Rotina de Corpo Inteiro, respectivamente) e 1RM_AGACHAMENTO_ (28,2% e 28,6% para Grupo Rotina Distribuída e Grupo Rotina de Corpo Inteiro, respectivamente) foram quase idênticas. O TE para 1RM_SUPINO_ (0,47 e 0,38 para Grupo Rotina Distribuídae Grupo Rotina de Corpo Inteiro, respectivamente) e 1RM_AGACHAMENTO_ (0,92 e 0,82 para Grupo Rotina Distribuída e Grupo Rotina de Corpo Inteiro, respectivamente) também foi muito semelhante entre os grupos. Esses achados refletem a literatura atual. Dados científicos sugerem que os benefícios da manipulação da frequência sobre o ganho de força são determinados pelo aumento do volume de treinamento. Quando o volume de treinamento é mantido constante, o aumento da frequência não parece proporcionar ganhos adicionais.^(^^10^^)^ Entretanto, a maioria dos estudos realizados até hoje empregou frequências de TF de 3 dias por semana por grupo muscular ou menos. Os achados deste estudo complementam a literatura corrente, uma vez que mostram que o TF realizado quatro vezes por semana não proporciona ganhos maiores do que o treinamento realizado duas vezes por semana.

Acredita-se que regimes de Rotina Distribuída aumentem a capacidade de treinar no nível máximo de esforço para uma dada intensidade, gerando maior esforço muscular em uma sessão específica de treinamento.^(^^11^^)^ Tais programas de treinamento facilitariam a recuperação, uma vez que a alternância de grupos musculares permite que um determinado músculo tenha mais tempo para se recuperar entre as sessões de treinamento. Entretanto, os resultados deste estudo sugerem que a Rotina Distribuída não induz maior resposta adaptativa em termos de força muscular em homens destreinados do que o treinamento Rotina de Corpo Inteiro, desde que o volume e a intensidade sejam iguais. Achados semelhantes foram relatados em estudos anteriores com homens com experiência em TF.^(^^12^^,^^13^^)^

No que se refere às adaptações hipertróficas, os programas de Rotina Distribuída e Rotina de Corpo Inteiro proporcionaram aumento semelhante da massa muscular nos membros superiores e inferiores. Acredita-se que o volume seja um fator importante na adaptação hipertrófica.^(^^14^^,^^15^^)^ Portanto, os achados relatados podem ter refletido o volume total equivalente de treinamento em ambos os programas de treinamento. Os resultados deste estudo são compatíveis com os de outros que compararam frequências de treinamento muscular de volume equalizado de, no mínimo, 2 dias por semana.^(^^16^^,^^17^^)^ No estudo de Brigatto et al.,^(^^18^^)^ baseado em medidas ultrassonográficas, frequências de treinamento de duas vezes por semana proporcionaram ganhos similares de hipertrofia em frequências de treinamento de quatro vezes por semana. Brigatto et al.,^(^^18^^)^ utilizaram regimes de Rotina Distribuída, enquanto este estudo comparou regimes de Rotina Distribuída e Rotina de Corpo Inteiro.

Zaroni et al.,^(^^19^^)^ também compararam os efeitos de regimes de Rotina Distribuída e Rotina de Corpo Inteiro sobre a adaptação neuromuscular em homens bem treinados ao longo de oito semanas de treinamento. No estudo em questão, os grupos musculares foram treinados uma (Distribuído) ou cinco vezes (Total) por semana, e os participantes executaram os mesmos exercícios com número semelhante de repetições em cada semana de treinamento, ao longo de todo o período experimental. Diferente deste estudo, frequências mais altas de treinamento foram associadas a efeitos hipertróficos potencialmente maiores nos indivíduos submetidos ao regime de Rotina Distribuída, no qual os músculos foram treinados apenas uma vez por semana (apesar do volume equalizado). O fato de a amostra estudada ter sido composta por indivíduos bem treinados (experiência em TF de 2,4 a 6,4 anos) pode explicar parcialmente as diferenças de resultados.

Estudos futuros comparando frequências diferentes de treinamento se fazem necessários para melhor compreensão do impacto da frequência de treinamento sobre o aumento da massa muscular no longo prazo.

Algumas limitações deste estudo devem ser indicadas. O período experimental foi de apenas oito semanas e não houve controle de dieta. Para o ganho de força muscular apresentado no presente estudo, deve ser considerada a influência das adaptações neurais, inclusive no perfil dos sujeitos. Apesar dos ganhos significativos de força e hipertrofia muscular em ambos os grupos, não fica claro se a extensão do período de treinamento traria resultados diferentes. A EM foi mensurada somente na porção média dos músculos. Essa região costuma ser considerada representativa do crescimento geral de um determinado músculo. Entretanto, acredita-se que a hipertrofia seja um fenômeno regionalizado, e ganhos maiores, às vezes, são detectados proximal e distalmente.^(^^20^^,^^21^^)^ Finalmente, os achados descritos se limitam a indivíduos jovens e saudáveis do sexo masculino e não podem ser extrapolados para outras populações, como adolescentes, mulheres e idosos. Frequências mais altas de TF podem não ser tão bem toleradas por esses indivíduos e antecipar a ocorrência do sobretreinamento (*overtraining*) quando combinadas com altas intensidades de treinamento. Pesquisas futuras se fazem necessárias para determinar as respostas induzidas pela frequência de treinamento com monitoramento de ingestão de calórica, bem como repetições controladas na população deste estudo. Além disso, em nosso estudo, o controle de volume foi feito equalizando o número de séries em concordância com recomendações de estudos anteriores.^(^^22^^)^ Porém, é possível considerar que equalizar a carga total levantada (número de séries x número de repetições x carga levantada) poderia promover desfechos diferentes. Dessa forma, mais estudos devem ser realizados para maiores esclarecimentos.

Os achados deste estudo têm implicações importantes, uma vez que podem auxiliar os treinadores na formulação de programas individualizados de treinamento, contribuindo para a adesão ao exercício. De fato, os resultados apresentados sugerem que os profissionais especializados em TF e condicionamento podem utilizar uma ampla gama de frequências para otimizar a força e a hipertrofia muscular com um determinado volume semanal. Este estudo foi planejado de forma a isolar os efeitos da frequência sobre a adaptação muscular, mantendo as demais variáveis de treinamento constantes. Dada a relação dose-dependente entre volume e adaptação muscular,^(^^15^^,^^23^^)^ frequências mais altas podem permitir que os praticantes suportem volumes maiores de treinamento, o que, por sua vez, pode resultar em maior ganho de força e hipertrofia muscular. Se o volume adicional de treinamento resultante do aumento da frequência seria ideal ou excessivo é algo a ser determinado individualmente.

### Implicações de ordem prática

Os programas de Rotina Distribuída e Rotina de Corpo Inteiro promoveram ganho semelhante de força e espessura muscular nos membros superiores e inferiores. Ambas as estratégias são igualmente eficazes em indivíduos destreinados durante a fase inicial do treinamento (oito semanas). Os achados deste estudo podem aumentar a adesão ao exercício, uma vez que permitem a adequação do programa de treinamento às necessidades individuais. Os profissionais especializados em treinamento de força e condicionamento podem utilizar uma ampla gama de frequências de treinamento para otimizar a força e a hipertrofia muscular com um determinado volume semanal.

## CONCLUSÃO

Os programas de treinamento distribuído e de corpo inteiro promoveram ganho semelhante de força e espessura muscular nos membros superiores e inferiores. Esses achados sugerem que ambas as estratégias são igualmente eficazes em promover adaptações musculares em indivíduos destreinados durante a fase inicial do treinamento (oito semanas).
